# Don’t Stop Your Journal

**Published:** 1888-12

**Authors:** 


DON'T STOP YOUR JOURNAL —It is a bad plan to stop a journal which for years you have been taking. Even though you decide to subscribe for another, don't drop THE RECORD, which like and old friend and companion, has visited your home for years, it may be, telling you all the interesting facts connected with the progress of the profession, and often giving you suggestions which, in times of difficulty and doubt, have greatly aided and comforted you—suggestions which will go with you through life and worth ten times the paltry sum which the RECORD costs you. Even though you may have decided to retire from the practice you should continue to take and read the RECORD. It will be a satisfaction to you to keep up with the progress of the profession after you have retired. As a matter of information and interest it will repay you, and should circumstances require you to go back to the practice, as will probably be the case, you will be up an readv to resume the duties of the profession.

